# Mixed Micellar Gel of Poloxamer Mixture for Improved Solubilization of Poorly Water-Soluble Ibuprofen and Use as Thermosensitive In Situ Gel

**DOI:** 10.3390/pharmaceutics16081055

**Published:** 2024-08-10

**Authors:** Namon Hirun, Pakorn Kraisit, Supaporn Santhan

**Affiliations:** Thammasat University Research Unit in Smart Materials and Innovative Technology for Pharmaceutical Applications (SMIT-Pharm), Faculty of Pharmacy, Thammasat University, Pathumthani 12120, Thailand; pakorn54@tu.ac.th (P.K.); kdoublee.kee@gmail.com (S.S.)

**Keywords:** amphiphilic copolymers, polymeric micelles, mixed micellar gels, poloxamer 407, poloxamer 403, ibuprofen

## Abstract

The aqueous solution of binary mixtures of amphiphilic copolymers is a potential platform for fabricating mixed polymeric micelles for pharmaceutical applications, particularly in developing drug delivery depots for a poorly water-soluble compound. This study fabricated and investigated binary mixtures of poloxamer 403 (P403) and poloxamer 407 (P407) at varying P403:P407 molar ratios to develop a vehicle for the poorly water-soluble compound, using ibuprofen as a model drug. The cooperative formation of mixed micelles was obtained, and the solubility of ibuprofen in the binary mixtures was enhanced compared to the solubility in pure water and an aqueous single P407 solution. The binary mixture with the P403:P407 molar ratio of 0.75:0.25 at a total polymer concentration of 19% *w*/*v* exhibited the temperature dependence of micellization and sol-to-gel characteristics of the thermosensitive mixed micellar gels. It possessed suitable micellization and gelation characteristics for in situ gelling systems. The release of ibuprofen from the thermosensitive mixed micellar depots was sustained through a diffusion-controlled mechanism. The findings can aid in formulating binary mixtures of P403 and P407 to achieve the desired properties of mixed micelles and micellar gels.

## 1. Introduction

Polymeric micelles are colloidal nanosystems of amphiphilic copolymers that self-assemble at or above their critical micellization concentration (CMC) [1]. The construction of the polymeric micelles consists of the hydrophobic core and the hydrophilic corona [2]. The hydrophobic micelle core can serve as a valuable reservoir for medicinal compounds with poor solubility in water, while the hydrophilic micelle corona can act as a protective barrier between the micelle core and the surrounding aqueous media. The copolymer structure is the primary factor determining micelle formation in amphiphilic copolymer solutions and the micellar solubilization of hydrophobic compounds in a certain aqueous environment. Apart from the capacity for solubilizing poorly water-soluble pharmaceuticals in the core, the supramolecular complexes of the polymeric micelles have the ability to prolong drug release, resulting in a dose reduction and a diminution in side effects [3]. In addition to the concentration-dependent self-organization behavior, the aqueous solutions of certain amphiphilic copolymers undergo a transition to micellar liquids as the temperature rises to a critical micellization temperature (CMT). As concentrations and/or temperatures increase, the interactions between the micellar assemblies can form a viscoelastic gel network whose unique gelation characteristics depend on the structural composition and molecular architecture of the amphiphilic copolymers [4]. The temperature at which the thermosensitive amphiphilic copolymer solution undergoes a liquid-to-gel transition is called the gelation temperature [5,6]. If the gelation temperature of the thermosensitive micellar gels is near body temperature, the vehicles can be delivered in a liquid state and then undergo gelation in situ after being applied to the body [6]. Developing thermosensitive micellar gels based on amphiphilic copolymers holds great promise for drug delivery applications.

Poloxamers, also known by the trade name Pluronic^®^, are a family of amphiphilic triblock copolymers with the basic chemical structure of poly(ethylene oxide)-poly(propylene oxide)-poly(ethylene oxide) (PEO-PPO-PEO). The polymeric members of the poloxamers family have attracted considerable attention in developing drug vehicles due to their unique surface activity, low toxicity, and minimal immune response [7,8,9]. Although the basic molecular architecture of the copolymer members in the poloxamers family is PEO-PPO-PEO, the number of ethylene oxide (EO) and propylene oxide (PO) units varies depending on the specific type of the amphiphilic copolymer [10]. These copolymers possess amphiphilic characteristics, and their hydrophilic-lipophilic balance (HLB) values are significantly affected by the quantity of EO and PO units [11]. Poloxamer 407 (P407) comprises a central hydrophobic block with 67 PO units and two terminal hydrophilic blocks, each with 98 EO units [12]. P407 possesses distinctively attractive attributes, including thermosensitivity and self-assembling ability [5]. Upon heating, P407 unimers at certain concentrations undergo micellization, followed by gelation due to the temperature-induced intermicellar packing [13]. To achieve thermosensitive gelation, a concentration of P407 greater than 15% is required [14]. The gelation temperature of the aqueous P407 solution is concentration-dependent; higher concentrations of P407 result in lower gelation temperatures [14,15]. For instance, it has been observed that the gelation temperature of 16% P407 in aqueous solution was approximately 29 °C, whereas the gelation temperature of 18% P407 in aqueous solution was around 25 °C [13]. Nevertheless, the gelation temperature of P407 is not appropriate for use as an in situ thermosensitive drug delivery since P407 generally transforms from a liquid to a gel at temperatures lower than the acceptable temperature range (30–36 °C) for administration under physiological conditions [16,17,18]. In addition, P407 is considered particularly hydrophilic due to its high HLB value of 22, which is attributed to a greater proportion of hydrophilic EO units relative to hydrophobic PO units [10,11].

Mixed micelles composed of distinct amphiphile systems that aggregate into micellar structures are scientifically and industrially intriguing [19,20]. This is because the characteristics of mixed micelles can be tailored to meet specific needs through simple composition variations rather than creating new materials through synthesis [19]. Binary mixtures of different amphiphilic copolymer surfactants can form mixed micelles through cooperative micellization of the two copolymers due to their favorable interactions or lead to the coexistence of two distinct populations of micelles [21]. Combining each polymeric component at varying molar ratios can achieve the desired micelle characteristics from the mixed micelles present in appropriate binary mixed-polymer systems [22]. Therefore, the selection of potential polymeric components as well as appropriate composition adjustment are important strategies for the development of mixed micelles. Combining distinct copolymers with a similar PPO chain length for binary mixed-polymer systems of poloxamers could cause cooperative interaction [20,23]. Poloxamer 403 (P403) has a central hydrophobic block composed of 67 PO units, similar to P407, but each of the two terminal hydrophilic blocks of P403 contains approximately 21 EO units [12]. The micellar solutions of P403 can exhibit thermosensitive gelation when the P403 concentration is greater than 20% [24]. The gelation temperature of the P403 solution depends on the polymer concentration. It has been reported that the liquid (sol)-to-gel transition cannot occur at temperatures lower than or equal to 37 °C when the P403 concentration was less than 28% [25]. P403, with its HLB of 8, is more hydrophobic than P407 and can be considered an efficient excipient for developing a vehicle for hydrophobic compounds. It was of interest to examine the binary mixed-polymer systems composed of P407 and P403. This could potentially lead to the formation of mixed micellar gel, which represents a promising candidate for pharmaceutical applications.

Ibuprofen, a non-steroidal inflammatory drug, was chosen as a model compound for pharmaceutical purposes. Ibuprofen is a poorly water-soluble drug, with an aqueous solubility of around 0.232 mM (0.048 mg/mL) at 25 °C and 0.324 mM (0.067 mg/mL) at 30 °C [26,27]. From a pharmaceutical development perspective, the solubility of the drug in the solvent should exceed 1 mg/mL in order to establish an efficient drug delivery system [28,29,30]. The development of injectable drug delivery systems, including injectable depots or implants, may be beneficial for ibuprofen delivery [31]. However, the poor solubility of this drug is a major problem for developing injectable dosage forms [32]. There has been a growing interest in developing formulation strategies for the fabrication of in situ gels that are capable of incorporating ibuprofen and delivering drug molecules into the desired body cavities [31,33,34,35]. The in situ forming depots containing ibuprofen hold promise for a variety of administration routes and applications, such as rectal administration for non-steroidal anti-inflammatory use in infants and children and periodontal administration for periodontitis treatment [31,33,34]. The strategy of solubilizing hydrophobic compounds in surfactant aggregates may address the challenges faced by drugs with low aqueous solubility in formulation development. The mixed micellar gels made from the PEO-PPO-PEO block copolymer chains may combine the advantages of micelle and thermosensitive gel systems for drug delivery.

In this study, the micellization and CMC of aqueous solutions of P407, P403, and binary mixed P403/P407 were examined using isothermal titration calorimetry (ITC). The synergistic or antagonistic interaction of the two copolymers at varying molar ratios was then explored. The solubilization of ibuprofen into single and mixed polymeric micellar solutions was examined. Tube tilting experiments were then conducted to screen the suitable total polymer concentration and P403:P407 molar ratio for a thermosensitive mixed micellar gel. For the thermosensitive mixed micellar gels with a suitable gelation temperature, temperature-induced micellization and gelation were also characterized. Furthermore, the in vitro release of ibuprofen from the mixed micellar gels was assessed.

## 2. Materials and Methods

### 2.1. Materials

P403 and P407 were supplied by Sigma-Aldrich (Saint Louis, MO, USA). Ibuprofen was obtained from PC Drug Co., Ltd. (Bangkok, Thailand). All other compounds used in this study were of analytical grade.

### 2.2. Sample Preparation

The aqueous solutions of P403, P407, and the binary mixtures of P403 and P407 were prepared using a ‘cold method’ as previously described [13]. The water used for sample preparation was Milli-Q water. P403 or P407 solution was prepared by dissolving the necessary amount of polymeric compound in half of the total water content. Cold water was then added to achieve the final desired volume of each sample. Each polymer solution was stirred continuously in an ice bath to obtain a clear solution. The binary mixtures of P403 and P407 were prepared for various total polymer concentrations with the P403:P407 molar ratios of 0.25:0.75, 0.5:0.5, and 0.75:0.25. The required amount of P403 was dissolved in half of the total water content, and then the required amount of P407 was dispersed into the previously prepared P403 dispersion. After that, the remaining cold water was added to the mixture to adjust the volume to the required final volume, and the binary mixture was stirred until it became homogeneous. The polymer samples containing ibuprofen were prepared by adding the necessary quantity of ibuprofen to the polymer dispersion at ambient temperature before adjusting the total volume of the sample to the final volume.

### 2.3. Isothermal Titration Calorimetry

Isothermal titration calorimetry (ITC) measurements were performed using a MicroCal PEAQ-ITC microcalorimeter (Malvern Panalytical, Malvern, UK) at 30 °C. The concentration of the amphiphilic polymer utilized as a titrant in the injection syringe must be greater than CMC [36,37]. The heat change during titration should be within an acceptable range, neither exceeding the measuring capacity nor being too low compared to background noise [36]. The total polymer concentration of 1.2 mM was chosen because it was suitable based on the above considerations. Titrations involved injecting one μL of 1.2 mM polymer solution—either single-polymer solution or binary polymer mixture—into the sample cell containing Milli-Q water for 38 injections at an interval of 120 s after a small initial injection was introduced with a volume of 0.4 μL. The experiments were conducted in triplicate. Data analysis was conducted with the MicroCal PEAQ-ITC Analysis Software version 1.41 and SigmaPlot 15.0 (Grafiti LLC, Palo Alto, CA, USA).

### 2.4. Dynamic Light Scattering

Dynamic light scattering (DLS) measurements were performed using a Malvern Zetasizer Nano ZS (Malvern Instruments, Malvern, UK) at 30 °C. To ensure that the polymer concentration was high enough to produce micelle formation without causing multi-scattering effects [4], the polymer concentration of 1.2 mM was used based on our preliminary study. The experiments were conducted in triplicate. The DLS data were processed using the Zetasizer software version 7.13.1.

### 2.5. Solubility Study

In each flat-bottom bottle, a sufficient quantity of ibuprofen powder (100 mg) was added to ensure supersaturation in 10 mL of water or polymer solution with a total polymer concentration of 1.2 mM. The bottles were shaken at a speed of 70 rpm for 24 h in a water bath at 30 °C; after that, the supernatant was removed and filtered through a syringe filter with a pore size of 0.45 μm [38,39]. The filtrate was diluted with ethanol, and the absorbance was determined using the UV-visible spectrophotometer (UV-1800, Shimadzu, Tokyo, Japan) at 264 nm [40]. The solubility investigation for each sample type was conducted in triplicate.

### 2.6. Test Tube Tilting Experiment

The test tube tilting method was applied to evaluate the sol-to-gel phase transition. Each test tube containing the sample was placed in a water bath and then equilibrated for 5 min at each temperature before the test tube was tilted to evaluate the flowability of the sample. The sol and gel states of each sample were evaluated in the temperature range of 20–40 °C with a temperature increment step of 1 °C. The sample was characterized as being in a gel state when it did not flow upon tilting the test tube [41].

### 2.7. Differential Scanning Calorimetry

Differential scanning calorimetry (DSC) measurements were performed in the temperature range of 5–45 °C with a heating rate of 1 °C/min under a nitrogen atmosphere using a Mettler Toledo DSC 3+ differential scanning calorimeter (Mettler-Toledo, France). Data were analyzed using the STARe Evaluation software version 16.40.

### 2.8. Rheometry

Temperature sweep rheological measurements were performed using a HAAKE MARS 40 rheometer (ThermoFisher Scientific, Bremen, Germany) within the linear viscoelastic region (LVR) predetermined by an amplitude sweep test for each sample. The amplitude sweeps were conducted at the lowest and highest temperatures (5 and 45 °C) in the temperature range used for temperature sweeps. Then, the temperature-dependent viscoelasticity was determined under controlled stress within the predetermined LVR [42]. A 60 mm parallel plate geometry with a 0.5 mm gap was used. Temperature sweep data were collected at an oscillatory frequency of 1 Hz in the temperature range of 5 to 45 °C with a temperature sweep rate of 1 °C/min. The experiments were performed in triplicate. Data were evaluated using the RheoWin Data Manager software version 4.87.0001.

### 2.9. In Vitro Release Study

The membrane-less diffusion method was employed to evaluate in vitro drug release, enabling direct contact between the gel and the release medium [6]. The in vitro release studies were conducted in a water bath shaker set at 37 °C and a shaking speed of 40 rpm [6,16]. A gel was formed by placing a 1 mL drug-loaded sample into a flat-bottomed bottle within the water bath shaker. Next, 15 mL of pre-warmed release medium (phosphate buffer, pH 7.4) was carefully transferred onto the gel surface. At predetermined intervals, 2 mL of release medium was drawn from the bottle and replaced with 2 mL of the fresh release medium. The ibuprofen amount in the withdrawn release medium was quantified using the UV-visible spectrophotometer (UV-1800, Shimadzu, Tokyo, Japan) at 222 nm [43].

The experimental data can be analyzed and fitted to a variety of mathematical drug release models in order to obtain insight into the mechanisms behind drug release characteristics [16,44,45,46]. The release data modeling was examined using the DDSolver program [47]. By analyzing the initial 60% of the release curve, the release exponent (*n*) was calculated in accordance with the Korsmeyer–Peppas equation (Equation (1)) to identify the release mechanism [6,16,44,45,48]. In addition to the Korsmeyer–Peppas equation, zero-order (Equation (2)), first-order (Equation (3)), and Higuchi (Equation (4)) models, which are practically applied for comparison and determining the best-fitting drug release model for the drug release profile of the drug-loaded hydrogels [44,45], were also utilized.

(1) $$F=K_{KP}\cdot t^{n}$$

(2) $$F=K_{0}\cdot t$$

(3) $$F=100\cdot \left[1-e^{-K_{1}\cdot t}\right]$$

(4) $$F=K_{H}\cdot t^{0.5}$$

where *F* is the fraction (%) of drug released in time *t*. *K_KP_*, *K*_0_, *K*_1_, and *K_H_* are Korsmeyer–Peppas release constant, zero-order release constant, first-order release constant, and Higuchi release constant, respectively.

### 2.10. Statistical Analysis

Means and standard deviations were derived from triplicates, and the data were subjected to statistical analysis using the analysis of variance (ANOVA). Tukey post hoc multiple comparisons were used to evaluate the differences between the sample groups at a significance level of 0.05.

## 3. Results and Discussion

### 3.1. Single and Mixed Polymeric Micelles

**Figure 1:**
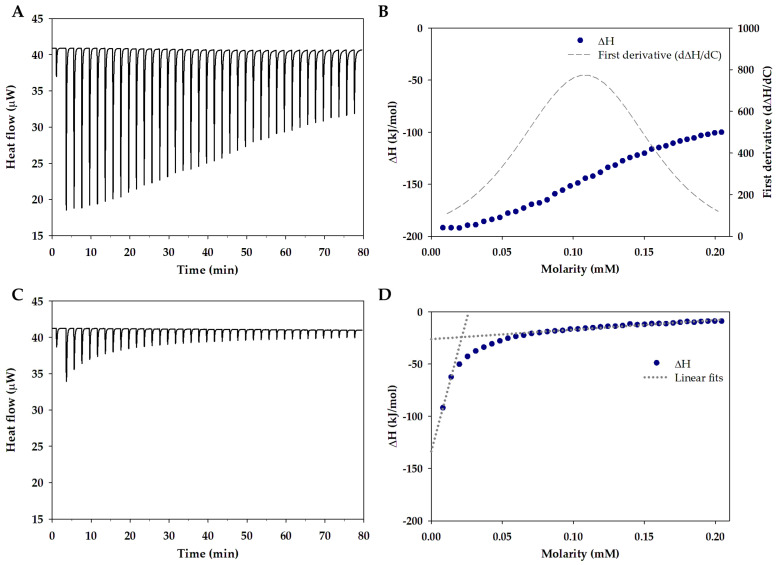
(**A**) Raw ITC thermogram of P407. (**B**) Normalized titration curve of P407. The dashed line represents the first derivative of the titration curve. (**C**) Raw ITC thermogram of P403. (**D**) Normalized titration curve of P403. The dotted lines represent the linear fits of the titration curve.

Isothermal titration calorimetry (ITC) is a powerful technique capable of monitoring heat discharge or consumption associated with self-assemble transition of surfactant molecules [7,36,37,49]. The self-assembly of single and mixed polymer surfactants into micelles was monitored using ITC. As is well known, the transformation of the dispersed unimers into micelles is induced by increasing the surfactant concentration to be greater than CMC. However, each surfactant has its own micellization characteristics. During the transformation from unimers to micelles or vice versa, heat is consumed or discharged [4]. Therefore, the self-assembly of polymer surfactants was investigated using ITC. Figure 1A represents the time evolution of the heat flow following consecutive injections of concentrated P407 into water. According to Figure 1A, the first few injections of the polymer solution produced large calorimetric signals representing the calorimetric signal characteristic of the pre-micellar phase [37]. For the first few injections, the titration of P407 micellar solution into water caused the dissociation of the micelles into the unimers. The calorimetric signal observed in the pre-micellar phase mainly corresponds to the micelle dissociation and the dilution of unimers [4,37]. After consecutive injections, the magnitude of the calorimetric signal gradually decreased because the elevated concentration of polymer surfactant within the sample cell prevented micelle dissociation. The last injections displayed calorimetric peaks of small and relatively comparable magnitudes, indicating the calorimetric characteristics of the post-micellar phase. In this phase, the surfactant concentration exceeded the CMC, and the dilution of the micelles was dominant [4,37].

Figure 1B shows the enthalpogram, the plot of integrated enthalpy changes per mole as a function of surfactant concentration, for P407. The analysis of the enthalpograms can be adopted to evaluate the CMC for the polymer surfactants. The physical properties and micellization characteristics of surfactants dictate the shape and profile of the curves. For the block copolymers composed of PEO and PPO blocks, sigmoidal and non-sigmoidal curves are the two primary forms of enthalpograms reported in studies [4,49,50]. When the enthalpograms exhibit the sigmoidal characteristic, the concentration at which the first derivative of the curve shows a maximum value represents the CMC value [4,50]. For the enthalpogram with a non-sigmoidal feature, the CMC can be estimated at the inflection point, which is the crossover between linear fits at the lowest and highest concentration regions of the curve [4,49]. From Figure 1B, it can be seen that the enthalpogram of P407 represented a sigmoidal shape. This pattern is consistent with results reported in other studies [50,51]. The first derivative of the raw data was computed, and the first derivative plot is also represented in Figure 1B.

The time evolution of the heat flow subsequent to consecutive injections of concentrated P403 into water is shown in Figure 1C, and the enthalpogram for P403 is presented in Figure 1D. The enthalpogram of P403 in Figure 1D exhibited a non-sigmoidal curve with a pronounced increase in the initial concentration range and the absence of a pre-micellar zone, suggesting that amphiphilic polymers have a relatively low CMC [4]. The CMC of P403 can be determined based on the reflection point, represented as the crossover of both dotted lines in Figure 1D.

**Figure 2:**
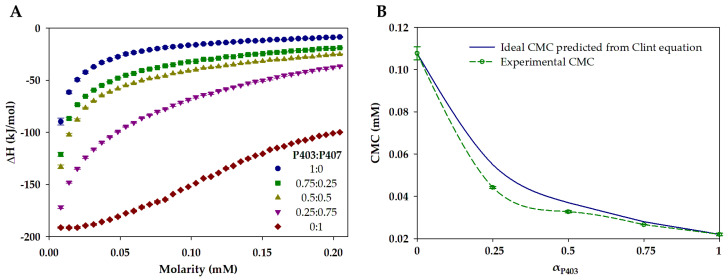
(**A**) Normalized titration curves of single P403 solution (the molar ratio of P403:P407 = 1:0), single P407 solution (the molar ratio of P403:P407 = 0:1), and binary mixtures of different molar ratios of P403:P407. (**B**) Plot of experimental CMC and ideal CMC predicted from Clint equation as a function of *α*_*P*403_.

Figure 2A shows the enthalpograms of binary mixtures of different molar ratios of P403 and P407 (the molar ratios of P403:P407 = 0.75:0.25, 0.5:0.5, and 0.25:0.75), comparing them to those of single-polymer systems. All enthalpograms for binary mixtures had non-sigmoidal curve characteristics. This indicated that the micellization behavior of binary mixtures was influenced by the presence of P403, even when the molar ratio of P403:P407 was low. Surfactants exhibiting distinct behaviors can mutually influence one other, particularly in the micellization of mixed micelles. The fabrication of polymer surfactant mixtures can result in performance attributes that combine or deviate from those of a single-polymer surfactant. The interactions of surfactants within the micelles are responsible for the performance attributes obtained. Mixing two distinct polymer surfactants in various compositions of each component can cause either ideal or non-ideal behavior of binary mixtures. It is well known that the fundamental estimation based on the Clint equation can be adopted to judge the ideality of the binary mixtures. The comparison of the experimental CMC value with the value of an ideal CMC (*CMC_ideal_*) calculated using the Clint equation is helpful for analyzing the ideal or non-deal behavior of binary mixtures. The values of *CMC_ideal_* for binary polymer mixtures of varying mole fractions (α) were derived from the Clint equation as shown below [37,52,53,54]:

(5) $$\frac{1}{{CMC}_{ideal}}=\frac{\alpha_{P403}}{{CMC}_{P403}}+\frac{\alpha_{P407}}{{CMC}_{P407}}$$

where *α*_*P*403_ and *α*_*P*407_ are the mole fractions of P403 and P407 in the total mixed solute. These mole fractions are on a surfactant-only basis; the mole fraction of each polymer surfactant was calculated by the ratio of the moles of the constituent to the total moles of both constituent polymer surfactants in the mixture [54,55]. Accordingly, the sum of *α*_*P*403_ and *α*_*P*407_ equals 1 [37,53]. The values of *α*_*P*403_ for the binary mixtures with the P403:P407 molar ratios of 0.75:0.25, 0.5:0.5, and 0.25:0.75 are 0.75, 0.5, and 0.25, respectively. In this equation, *CMC*_*P*403_ and *CMC*_*P*407_ are the CMC values of the aqueous solution of single P403 and P407, respectively.

Figure 2B represents the plots of the experimental CMC values and the ideal CMC values as a function of the mole fractions of P403. The average values of experimental CMC for single P407 solution and single P403 solution were 0.108 and 0.022 mM, respectively, falling within the range of the CMC values reported in the literature for each of these single-polymer systems [49,56,57]. The average experimental CMC values for the binary mixtures with the P403:P407 molar ratios of 0.75:0.25, 0.5:0.5, and 0.25:0.75 were 0.027, 0.033, and 0.044 mM, respectively. It can be seen from Figure 2B that the experimental CMC values deviate from the ideal CMC values for the binary mixtures at all investigated mole fractions. The deviation of the experimental CMC values from the ideal CMC values signifies the non-ideal behavior associated with the interaction between the amphiphiles. The interactions between the amphiphiles could be either synergistic or antagonistic. The existence of the antagonistic interaction is denoted by a positive divergence when the experimental CMC value is higher than the ideal CMC value [58]. On the other hand, the synergistic interaction is signified by a negative deviation when the experimental CMC value is lower than the ideal CMC value [58]. As shown in Figure 2B, the experimental CMC values have a negative deviation with respect to the ideal CMC values for all mole fractions of the binary mixtures. The experimental CMC values were lower than the ideal CMC values, implying substantial synergism in the mixed micelle formation [58]. Furthermore, the synergism in the mixed micelle formation can be evaluated using the interaction parameter (*β*), which can be calculated using the equation given below [58,59].

(6) $$\beta =\frac{1}{{\left(1-X_{P403}\right)}^{2}}ln\frac{\alpha_{P403}{CMC}_{mix}}{X_{P403}{CMC}_{P403}}$$

where *CMC_mix_* is the experimental CMC value of the binary mixture, and *X*_*P*403_ is the micellar composition of P403 in the mixed micelle. The value of *X*_*P*403_ can be estimated by iteratively solving the following equation [58]:

(7) $$\frac{{X_{P403}}^{2}ln\left(\frac{\alpha_{P403}{CMC}_{mix}}{X_{P403}{CMC}_{P403}}\right)}{{\left(1-X_{P403}\right)}^{2}ln\left(\frac{\left(1-\alpha_{P403}\right){CMC}_{mix}}{\left(1-X_{P403}\right){CMC}_{P407}}\right)}=1$$

**Figure 3:**
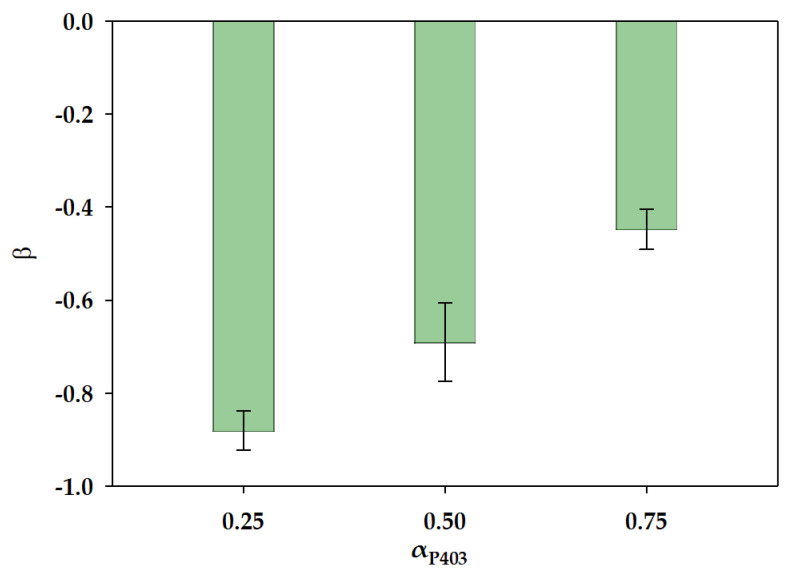
Plot of *β* as a function of *α*_*P*403_ for the binary mixtures.

**Scheme 1:**
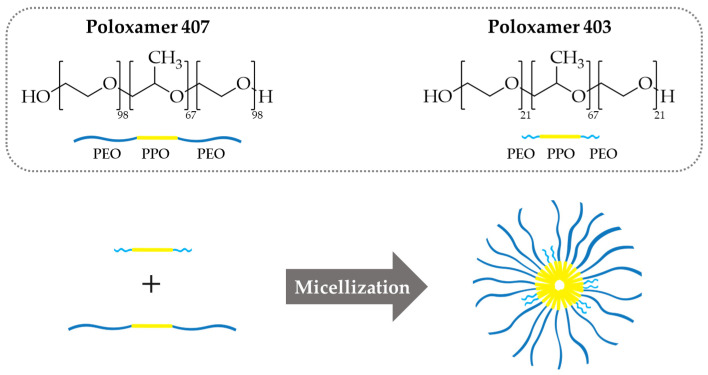
Schematic representation of the formation of mixed polymeric micelle.

Figure 3 shows the value of the *β* parameter for the binary mixture containing each mole fraction of P403. According to the results, the mean values of the *β* parameters show negative values, ranging from −0.45 to −0.88 over the entire mole fraction range. The deviation of the *β* parameter value from zero is attributed to interactions among the amphiphiles. The positive deviation from zero signifies a repulsive (antagonistic) interaction, while a negative deviation implies an attractive (synergistic) interaction between two amphiphiles [58,59]. The negative values of the *β* parameter in this study indicate the favorable attraction between P403 and P407 in the mixed micelles. This synergism leads to the favorable formation of micellar aggregates, which is beneficial for pharmaceutical formulation. The negative *β* parameter value of binary polymer mixtures verifies the interaction capability, thereby promoting drug solubility within the lipophilic core of the mixed micelles [60]. It has also been reported that compounds of P407 and nonionic surfactant in pharmaceutical formulations exhibit a moderate synergistic effect with the interaction parameter, ranging from −0.22 to −1.54 [59]. The binary mixtures of P403 and P407 at all investigated mole fractions showed favorable interactions between the components [59,61]. Hydrophobic interactions between the PPO blocks formed the association among distinct polymer chains, while the PEO blocks were oriented toward the outside and facing the aqueous environment [62]. This led to the formation of aggregates as mixed polymeric micelles with the hydrophobic core and the hydrophilic corona, as presented in Scheme 1.

**Figure 4:**
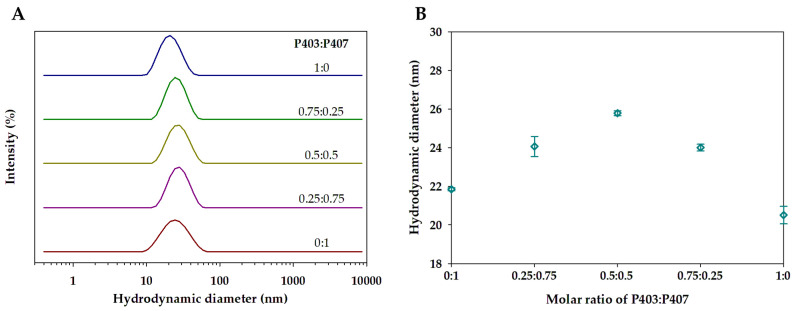
(**A**) The intensity-weighted hydrodynamic radius distribution measured by DLS of single P403 solution (the molar ratio of P403:P407 = 1:0), single P407 solution (the molar ratio of P403:P407 = 0:1) and binary mixtures of different molar ratios of P403:P407. (**B**) Plot of hydrodynamic diameter as a function of molar ratios of P403:P407.

To examine the impact of combining P407 and P403 at different molar ratios on the aggregation of micelles at the nano-scale, dynamic light scattering (DLS) analyses were conducted on single-polymer solutions and binary mixtures containing P403 and P407 at various molar ratios. The intensity-weighted distribution of the hydrodynamic diameter of single P407 micellar solution, single P403 micellar solution and the solutions of binary mixtures containing P403 and P407 at various molar ratios is shown in Figure 4A. Both single-polymer surfactant solutions, P403 and P407, display unimodal curves. All binary mixtures also exhibited a unimodal size distribution, reflecting the formation of uniform mixed aggregates [63]. This may be attributed to the association between P407 and P403 copolymers, which formed mixed micelles with a single-size distribution [7]. The average hydrodynamic diameters of micelles in single-polymer systems and binary mixtures at different molar ratios of P403 and P407 are shown in Figure 4B. The diameter of the micelles in the single P403 system is approximately 20.50 nm, which falls within the range of the mean size reported in the literature [64]. For the micelles in the single P407 system, the average hydrodynamic diameter is 21.84 nm, which is in general agreement with the previously reported sizes [65]. The average size value of the micelles in each binary mixture is slightly greater than the hydrodynamic diameters of the micelles found in both single-polymer systems. An increase in the size of micelles has also been reported for cooperative micellization through favorable interactions in binary mixtures of poloxamer 235 and P403 [21,66]. The mixed micelles present in binary mixtures may be larger than those of the parent polymers due to the formation of mixed micelles with a higher association number [66]. Certain compositions of binary mixtures achieved the higher packing density of the block copolymer chains, which led to the cooperative formation of mixed micelles with a higher association number [21,66].

### 3.2. Solubilization of Ibuprofen in Single and Mixed Polymeric Micelles

The solubility of ibuprofen in pure water at 30 °C observed in this study was 0.321 ± 0.015 mM, which compared well with the solubility of 0.324 mol/m^3^ (0.324 mM) reported in the literature [27]. To evaluate the drug solubilization efficiency of the aqueous solutions of surfactants, the drug solubility in pure water (*S_w_*) and the drug solubility in the aqueous solutions of polymer surfactants (*S_s_*) were used to calculate the value of the solubility enhancement (*SE*) by using following relation [67,68,69]:

(8) $$SE=\frac{S_{s}}{S_{w}}$$

**Figure 5:**
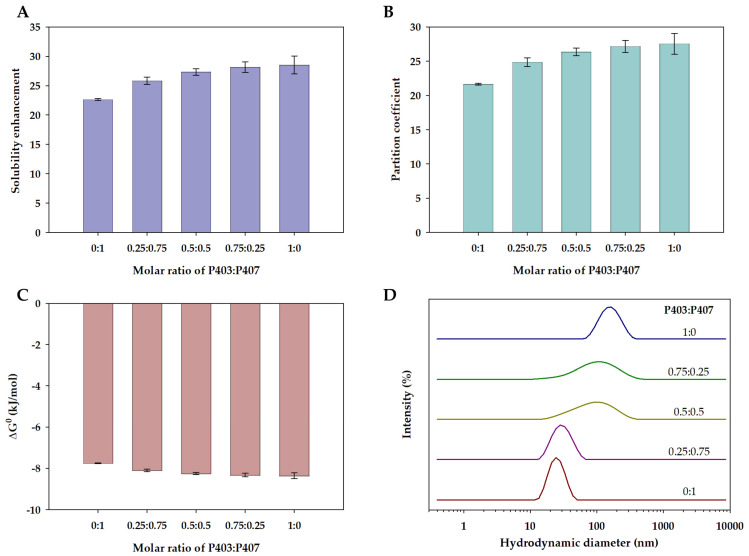
(**A**) Solubility enhancement, (**B**) partition coefficient, and (**C**) standard free energy of solubilization plotted as a function of molar ratios of P403:P407. (**D**) The intensity-weighted hydrodynamic radius distribution measured by DLS of the samples with different molar ratios of P403:P407 after ibuprofen solubilization.

Figure 5A illustrates the values of the solubility enhancement, which reflect the magnitude of the enhancement of drug solubility in the presence of polymeric micelles in water relative to drug solubility in pure water. All investigated polymer systems have solubility enhancement values greater than 1. The mean value of the solubility enhancement for the single P407 system is about 22-fold, and the solubility enhancement values for the single P403 system and the binary mixture systems are higher than 22-fold. This indicates that the presence of these polymeric micelles enhances the solubility of ibuprofen.

To better understand the insight into the solubilization of ibuprofen in the micelles, the micelle–water partition coefficient and the standard free energy of solubilization were estimated. For a certain concentration of surfactant, the micelle–water partition coefficient (*P*) represents the ratio between the drug concentration in the micelle and that in water and can be obtained using the following relation [70]:

(9) $$P=\frac{S_{s}-S_{w}}{S_{w}}$$

The solubilization resulting from micellization can be regarded as a partitioning of the drug between micelle and aqueous phases [70,71], and the standard free energy of solubilization (Δ*G*^0^) can be calculated from the partition coefficient value using the following equation [70]:

(10) $$\Delta G^{0}=-RTlnP$$

where *R* and *T* are the gas constant and the temperature in Kelvin unit, respectively.

Figure 5B represents the partition coefficient for single-polymer systems and binary mixtures. The partition coefficient values for all binary mixtures and the single P403 system were significantly higher than that of the single P407 system (*p* < 0.05). A greater value of the partition coefficient indicates a greater capacity of drug molecules to distribute within the micelles of the single P403 system and the binary mixtures compared with the single P407 system, as the more hydrophobic microenvironment of micelles is favorable for solubilized ibuprofen [70]. Although the mean value of the partition coefficient for the single P403 system is slightly higher than the mean values found for the binary mixtures, the difference among the single P403 system and the binary mixtures with P403:P407 molar ratios of 0.5:0.5 and 0.75:0.25 is not statistically significant. The Δ*G*^0^ values for single-polymer systems and binary mixtures are depicted in Figure 5C. The negative values of Δ*G*^0^ reflected the spontaneity of the solubilization and the transfer of the drug to the micellar phase [70]. The Δ*G*^0^ values of mixtures are significantly more negative than that of the single P407 system (*p* < 0.05), suggesting that enhancing the hydrophobic nature of the binary mixtures favors the spontaneous solubilization and promotes partition of ibuprofen into the mixed micelles [70,71]. The alteration in colloidal particle size may serve as a trace of drug molecule incorporation within the micelles [72]. To illustrate the size of the micelles after the solubilization of ibuprofen, the intensity-weighted distribution profiles of ibuprofen-loaded micelles are displayed in Figure 5D. Although the intensity-weighted distribution profiles of ibuprofen-loaded mixed micelles remained the unimodal curve characteristic, the hydrodynamic diameters of ibuprofen-loaded mixed micelles (Figure 5D), especially for the binary mixtures with P403:P407 molar ratios of 0.5:0.5 and 0.75:0.25, were larger than those of unloaded micelles (Figure 4A). The size increment confirmed the infiltration of drug molecules into the micelles [72]. The drug compound has solubilized in the micelles, resulting in the swelling of the micellar particle [70]. After drug solubilization, the average values of the polydispersity index (PDI) of single P407 and single P403 systems were 0.038 and 0.110, respectively. The average PDI values for the ibuprofen-loaded binary mixtures with the P403:P407 molar ratios of 0.75:0.25, 0.5:0.5, and 0.25:0.75 were 0.281, 0.272, and 0.097, respectively. The PDI value of 0.3 or below is indicative of acceptable narrow polydispersity, suggesting that the micelle population is homogeneous [73,74,75]. Although the ibuprofen-loaded binary mixtures with the P403:P407 molar ratios of 0.75:0.25 and 0.5:0.5 had a wider size distribution and a higher PDI than the single-polymer systems, PDI values less than 0.3 suggested that these mixed micelle systems were homogeneous and uniform [74].

### 3.3. Thermosensitive Micellization and Gel Formation

**Figure 6:**
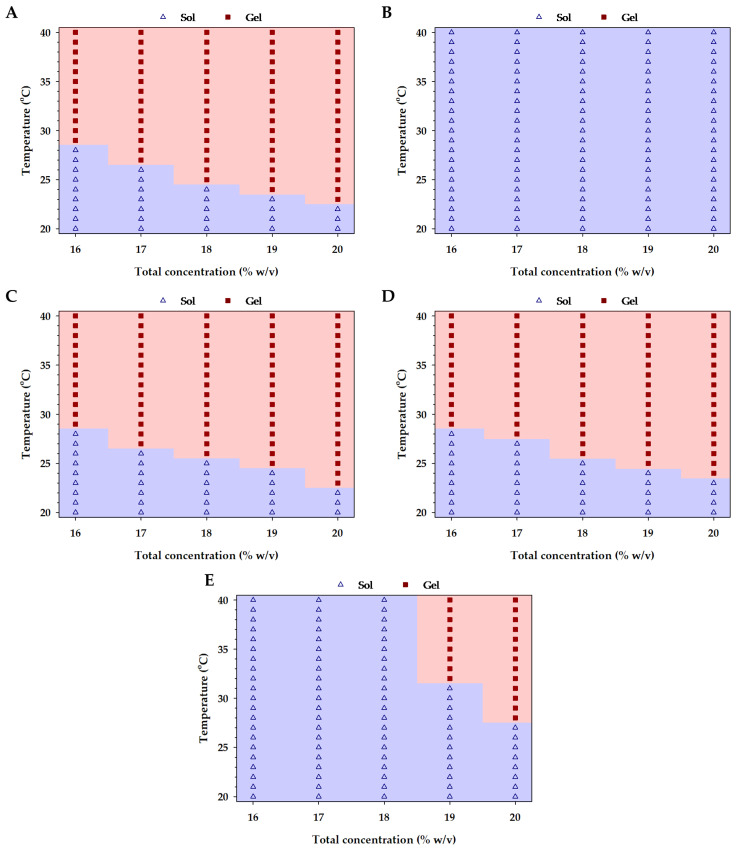
Sol-to-gel phase diagrams of (**A**) single P407, (**B**) single P403, and the binary mixtures at the P403:P407 molar ratios of (**C**) 0.25:0.75, (**D**) 0.5:0.5, and (**E**) 0.75:0.25.

In order to investigate the possible application of polymeric micelles as thermosensitive hydrogels for physiological purposes, the tube tilting experiments were conducted in the temperature range of 20–40 °C. Figure 6 displays the sol-to-gel phase diagrams that depict the sol and gel states in relation to the total polymer concentration and temperature. All examined P407 concentrations gelled within the investigated temperature range for the single P407 systems (Figure 6A). However, for single P407 systems, all samples became gel at temperatures below 30 °C, making their handling difficult for physiological application. The samples remained liquid for the single P403 system (Figure 6B) throughout the investigated concentration and temperature range. Our findings are in line with earlier research demonstrating that the sol-to-gel transition cannot occur at temperatures less than or equal to 37 °C when the P403 concentration is below 28% [25]. Figure 6C–E display the sol-to-gel phase diagrams of the binary mixtures with the P403:P407 molar ratios of 0.25:0.75, 0.5:0.5, and 0.75:0.25, respectively. When the total amount of polymers is 19–20% *w*/*v*, binary mixtures of P403 and P407 with molar ratios of 0.75:0.25 (Figure 6E) can raise the temperature at which the sol-to-gel transition happens compared to single P407 systems (Figure 6A) with the same total concentration of polymer. However, only the binary mixtures with P403:P407 molar ratio of 0.75:0.25 at a total polymer concentration of 19% *w*/*v* formed a gel at approximately 32 °C, which falls within the desired temperature range between 30 and 36 °C. Therefore, the binary mixture with the P403:P407 molar ratio of 0.75:0.25 at a total polymer concentration of 19% *w*/*v* was the potential candidate for thermosensitive hydrogel for physiological applications. Based on these results, the 19% *w*/*v* binary mixture with the P403:P407 molar ratio of 0.75:0.25 was used in further experiments, and this formulation was referred to as 19% 0.75P403–0.25P407.

**Figure 7:**
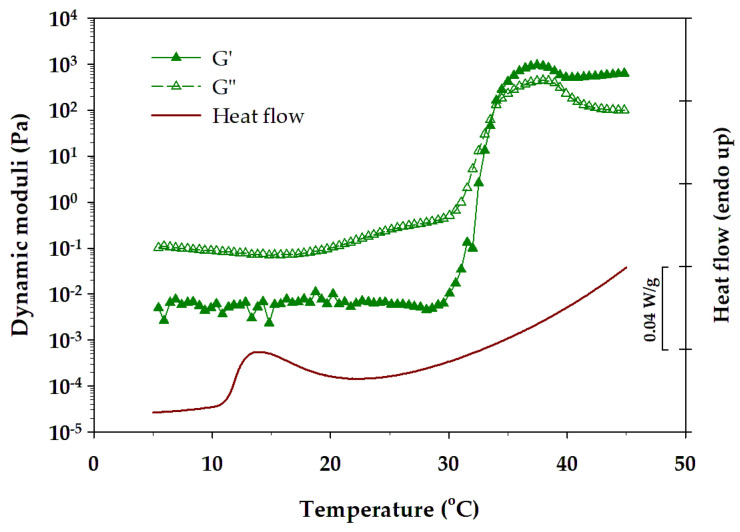
Storage modulus (G′), loss modulus (G″), and DSC heat flow as a function of temperature for 19% 0.75P403-0.25P407.

The thermosensitive micellization of 19% 0.75P403-0.25P407 was evaluated using differential scanning calorimetry (DSC). As shown in Figure 7, a single broad endothermic peak observed for the DSC heat flow curve of 19% 0.75P403-0.25P407 can be attributed to micellization [8]. Zhang et al. (2013) reported the observation of two endothermic peaks in mixtures of two distinct thermosensitive triblock copolymers [21], and the presence of two endothermic peaks may reflect the occurrence of separate micellization for each triblock copolymer [8,21]. However, the distinct two endothermic peaks reflecting separate micellization events were not detected in the binary mixture of P403 and P407 in this study, and the observed single broad endotherm could be indicative of the formation of the cooperative mixed micelles composed of P403 and P407 upon heating. This finding supports the assumption that the favorable interactions in binary mixtures of P403 and P407 participated in cooperative micellization.

The thermosensitive gelation of 19% 0.75P403-0.25P407 was characterized by measuring its temperature-dependent viscoelasticity, and the temperature dependence of the storage (G′) and loss (G″) moduli was also displayed in Figure 7. The sample of 19% 0.75P403–0.25P407 exhibited a change in viscoelasticity that was sensitive to an increase in temperature. When the temperature was low, the sample exhibited solution-like behavior (G″ > G′). When heated, the sample transformed into a gel (G′ > G″). As the temperature continued to rise near the sol-to-gel transition, G′ and G″ increased. Both dynamic moduli then reached the crossover point, where G′ equals G″, indicating the sol-to-gel transition caused by the temperature-induced intermicellar entanglements [13]. The gelation temperature obtained from the temperature sweep rheological experiment can be defined by the temperature at which G′ and G″ crossover occurs. The value of the gelation temperature of 19% 0.75P403-0.25P407 was 32.7 ± 1.2 °C, which was consistent with the result obtained from the tube tilting experiment.

### 3.4. Mixed Micellar Gels with Incorporated Ibuprofen

**Figure 8:**
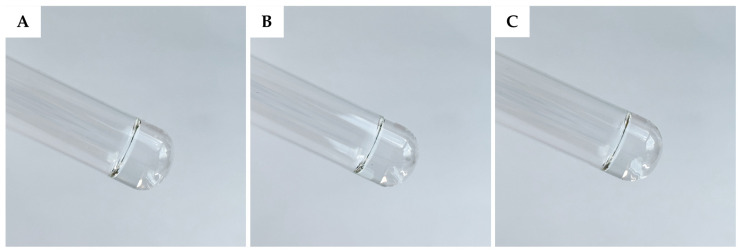
Digital photos of 19% 0.75P403-0.25P407 containing (**A**) 1.0 mg/mL, (**B**) 1.4 mg/mL, and (**C**) 1.8 mg/mL of ibuprofen at 37 °C.

**Figure 9:**
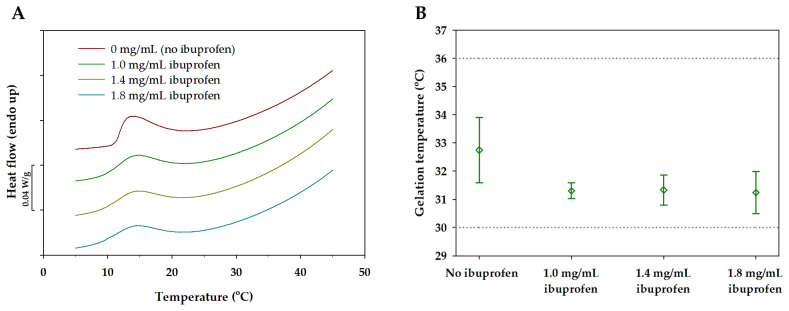
(**A**) DSC traces for plain 19% 0.75P403-0.25P407 (no ibuprofen) and 19% 0.75P403-0.25P407 containing 1.0 mg/mL, 1.4 mg/mL, and 1.8 mg/mL of ibuprofen. (**B**) The gelation temperature defined by the temperature sweep rheological measurements for plain 19% 0.75P403-0.25P407 (no ibuprofen) and 19% 0.75P403-0.25P407 containing 1.0 mg/mL, 1.4 mg/mL, and 1.8 mg/mL of ibuprofen. An acceptable gelation temperature range of 30–36 °C is indicated by the dotted lines.

Ibuprofen was incorporated into 19% 0.75P403-0.25P407 to obtain the samples with three different drug concentrations: 1.0 mg/mL (~4.85 mM), 1.4 mg/mL (~6.79 mM), and 1.8 mg/mL (~8.73 mM). The 1.0 mg/mL drug concentration represented the practical concentration of ibuprofen in acidic or salt form incorporated into the developed gel systems [35,76,77]. The concentration effect of the incorporated drug at higher concentrations of 1.4 mg/mL and 1.8 mg/mL was investigated, and it was found that the preparations of these samples were homogenous due to the improved solubilization of the drug in the binary polymer mixture. At simulated body temperatures, the mixed micellar gels of all these binary P403/P407 systems with the incorporated drug were homogenous and clear, as shown in Figure 8. The thermosensitive micellization and temperature-dependent gelation of 19% 0.75P403-0.25P407 with incorporated ibuprofen were also evaluated. As shown in Figure 9A, the single endothermic peak was also observed for each 19% 0.75P403-0.25P407 with incorporated ibuprofen. The endothermic peaks of the samples with drug incorporation appeared in the same temperature region observed for the sample with no drug incorporation. However, the samples containing ibuprofen showed a decrease in the height of the endothermic peaks, indicating a facilitation of micellization [13]. The gelation temperature marginally shifted to a lower temperature with the incorporation of ibuprofen into the binary polymer mixture, as shown in Figure 9B. The average and standard deviation values of the gelation temperature of 19% 0.75P403-0.25P407 were 32.7 ± 1.2 °C, while the gelation temperature values of 19% 0.75P403-0.25P407 with 1.0 mg/mL, 1.4 mg/mL, and 1.8 mg/mL of ibuprofen were 31.3 ± 0.3, 31.3 ± 0.5, and 31.2 ± 0.7 °C, respectively. However, these values were still within the acceptable gelation temperature range.

**Figure 10:**
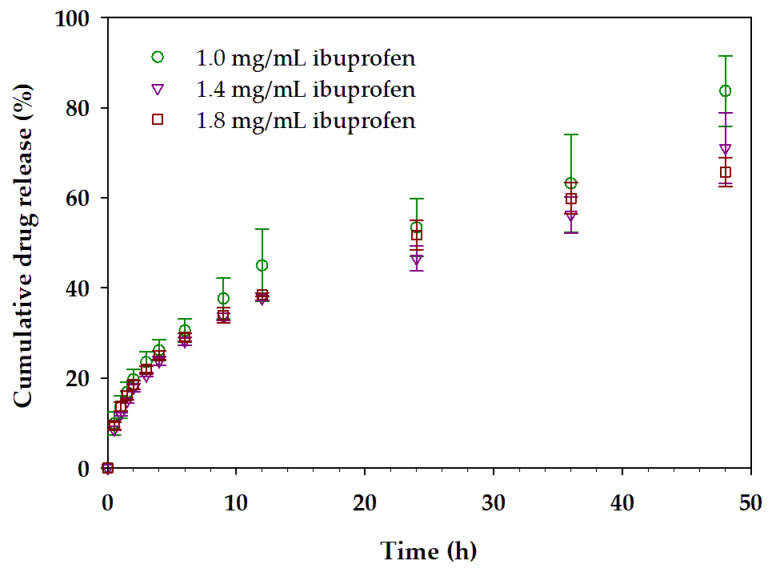
The cumulative drug release profiles of 19% 0.75P403-0.25P407 containing 1.0 mg/mL, 1.4 mg/mL, and 1.8 mg/mL of ibuprofen.

Figure 10 illustrates the in vitro release profiles of ibuprofen-loaded gel samples. All gel depots sustained the release of the drug molecules for 48 h. There are slight differences in the release behavior of three drug-loaded gel systems. Over 48 h, the mixed micellar gel sustained drug release, resulting in mean cumulative releases of approximately 84, 71, and 66% for 19% 0.75P403-0.25P407 with 1.0 mg/mL, 1.4 mg/mL, and 1.8 mg/mL of ibuprofen, respectively. The release data were fitted to various mathematical drug release models. The best-fit model was established using the coefficient of determination (R^2^) from release data modeling [45]. For all sample types, the R^2^ values obtained from fitting the release data with the zero-order and first-order models were low, ranging from 0.0081 to 0.4079 for the zero-order model and from 0.4376 to 0.6462 for the first-order model. The R^2^ values from release data modeling of the Higuchi model were 0.9926, 0.9525, and 0.9699 for 19% 0.75P403-0.25P407 with 1.0 mg/mL, 1.4 mg/mL, and 1.8 mg/mL of ibuprofen, respectively. The Korsmeyer–Peppas model demonstrated the best fit for all samples, with the highest R^2^, as presented in Table 1. According to the calculation based on the Korsmeyer–Peppas model, the release exponent ‘*n*’ values for all three gel formulations were less than 0.5 (Table 1). This suggests that the release of ibuprofen from these mixed micellar gel matrices is dominated by Fickian diffusion [48,78].

## 4. Conclusions

The synergistic assembly of mixed micelles was achieved, and the binary mixtures of P403 and P407 resulted in an increase in the solubility of ibuprofen in the mixed micellar vehicles compared to drug solubility in pure water and a solution containing P407. The binary mixture, with a molar ratio of 0.75:0.25 for P403:P407 and a total polymer concentration of 19% *w*/*v*, demonstrated thermosensitive micellization and gelation, which led to the formation of the thermosensitive mixed micellar gel. The mixed micellar gel possessed the appropriate gelation temperature, making it suitable for use as an in situ gelling system. These mixed micellar gels sustained the release of ibuprofen, with the primary mechanism being diffusion-controlled. The results of this study can be further utilized in refining the development of amphiphilic polymer mixtures to accomplish the desired properties of mixed micelles and micellar gels.

## Figures and Tables

**Table 1:** Release parameters for 19% 0.75P403-0.25P407 with loaded ibuprofen according to the Korsmeyer–Peppas model.

Concentration of Loaded Ibuprofen (mg/mL)	Korsmeyer–Peppas Model		
	*n*	*K_KP_*	R^2^
1.0 mg/mL	0.469	13.715	0.9960
1.4 mg/mL	0.409	13.148	0.9916
1.8 mg/mL	0.419	13.634	0.9991

## Data Availability

The original contributions presented in this study are included in the article; further inquiries can be directed to the corresponding author.
